# The differentiation courses of the Tfh cells: a new perspective on autoimmune disease pathogenesis and treatment

**DOI:** 10.1042/BSR20231723

**Published:** 2024-01-31

**Authors:** Qingya Yang, Fang Zhang, Hongyi Chen, Yuman Hu, Ning Yang, Wenyan Yang, Jing Wang, Yaxu Yang, Ran Xu, Chao Xu

**Affiliations:** 1 Division of Rheumatology, Affiliated Hospital of Integrated Traditional Chinese and Western Medicine, Nanjing University of Chinese Medicine, Nanjing, Jiangsu, 210028, China; 2 Division of Rheumatology, Jiangsu Province Academy of Traditional Chinese Medicine, Nanjing, Jiangsu 210028, China; 3 Division of Rheumatology, People’s Hospital of Mianzhu, Mianzhu, Sichuan, 618200, China

**Keywords:** autoimmune disease, differentiation courses, follicular helper T cells

## Abstract

The follicular helper T cells are derived from CD4^+^T cells, promoting the formation of germinal centers and assisting B cells to produce antibodies. This review describes the differentiation process of Tfh cells from the perspectives of the initiation, maturation, migration, efficacy, and subset classification of Tfh cells, and correlates it with autoimmune disease, to provide information for researchers to fully understand Tfh cells and provide further research ideas to manage immune-related diseases.

Follicular helper T cell (Tfh) is a subset of CD4^+^T cells, derived from the differentiation of naïve CD4^+^T cells. In 2000, the CXCR5^+^CD4^+^T cell subset in follicles of human tonsil tissue was found. This cell subset participates in the immune response in human tonsil tissue and promotes B cells to produce antibodies and immunoglobulins, so it is named B follicular helper T cells [1]. In 2009, It was proved that the transcription factor B-cell lymphoma-6 (Bcl-6) is an important factor for the production of Tfh cells in mice and designated Tfh cells lineage typing, which proved that Tfh cells are a special helper T cell (Th) cell subgroup. Tfh cells surface markers include chemokine receptor type 5 (CXCR5), programmed death-1 (PD-1), inducible co-stimulator (ICOS), CD40 ligand (CD40L), etc. These markers are related to the Tfh cells’ function [2]. This article reviews the differentiation, maturation, and effector stages of Tfh cells, as well as their relationship with autoimmune disease (AID).

## Early stage of Tfh cells differentiation

The process of naïve CD4^+^T cells differentiating into Tfh cells mainly occurs in the T cell region of lymphoid tissue, and is jointly regulated by immune cells such as dendritic cells (DCs), transcription factors such as Bcl-6, and cytokines such as achaete-scute homologue 2 (Ascl2).

### DC acts on early differentiation of Tfh cells through signal pathways such as IL-6/STAT3 and IL-12/STAT4

DCs and B cells both produce interleukin-6 (IL-6). After the combination of IL-6 and IL-21, the signal transducer and activator of transcription 3 (STAT3) could be activated to start Tfh cells differentiation [3].

In the lungs of asthmatic mice, conventional type 2 dendritic cells (cDC2) is the main DC subset responsible for Tfh cells differentiation. cDC2 produced a large amount of IL-6 and IL-21, which can more strongly induce the differentiation of Bcl-6-producing Tfh cells. Block the expression of IL-6, then the differentiation of Tfh cells was inhibited [4]. Compared with healthy people, Tfh cells expression level in rheumatoid arthritis (RA) patients was increased, and Tfh cells proportion was positively correlated with disease activity score in 28 joints (DAS28), phosphorylated STAT3 (pSTAT3) -Tyr705, and plasma IL-6 concentration. IL-6 induced STAT3’ Tyr705 phosphorylation, resulted in the overactivation of STAT3, and then drove Tfh cells differentiation [5]. This indicates the important role of the IL-6-pSTAT3-Tfh cells immunomodulatory axis in the pathological process of RA.

In inflammatory models, the induction of optimal Tfh cells differentiation and B-cell response requires the involvement of IL-6 [6]. Type I interferon (IFN-I) can up-regulate and increase the expression of CD86 on the surface of DCs and B cells, mediating the production of IL-6 by DCs, thereby enhancing the differentiation of Tfh cells [7]. Toll-like receptors (TLRs) specifically recognize pathogens and endogenous pro-inflammatory factors and trigger adaptive immune and inflammatory responses. Cytosine-phosphodiester-guanine (CpG)-B oligonucleotides belong to TLR ligands. The addition of CpG-B oligonucleotides to vaccine adjuvants can promote the production of IL-6 by DCs, which in turn enhances antigen-specific Tfh cells differentiation *in vivo* and promotes T cell-dependent B-cell responses [8]. Cutaneous DCs expand CXCR5^+^Tfh cells through IL-6 and IFN- α/β receptor non-dependent mechanisms, providing new ideas for vaccination [9]. Transcription factor T-cell factor 1 (TCF-1) is important for early T-cell development. TCF-1 promoted IL-6 receptor expression and increased the responsiveness of naïve CD4^+^T cells to Tfh cells signaling, and then promoted early Tfh cells differentiation by mediating the sustained expression of IL-6 receptor α (IL-6Rα) and IL-6/IL-6Rα downstream signal anti-IL-6Rβ: glycoprotein130 (gp130) [10]. The IL-6/STAT3 pathway promotes Thymocyte selection-associated high mobility group box 2 (Tox2) expression, while Tox2 inversely enhances IL-6 signaling and promotes Tfh cells differentiation [11]. The B lymphocyte-inducible maturation protein 1 (Blimp-1) is encoded by PR Domain Containing Protein 1 (*PRDM1*) [12]. In female mice, Blimp-1 deficiency led to more IL-6 secretion by DCs, promoted Tfh cells differentiation and germinal center (GC) responses, and produced lupus-like autoantibodies [13]. Total glucosides of paeony (TGP) improved symptoms and bone destruction in collagen-induced arthritis (CIA) model mice, and the expression of Tfh cells, IL-6 and IL-21, as well as STAT3 signaling pathways were also inhibited. It is speculated that TGP’s anti-arthritic effect is due to its inhibition of spleen Tfh cells differentiation and GC formation through the IL-6-STAT3 signaling pathway, thereby suppressing autoimmune responses [14]. Niclosamide is a potent STAT3 signaling inhibitor. In systemic lupus erythematosus (SLE) model mice, Niclosamide significantly reduced IL-6, p-STAT3, and TCF-1 levels, down-regulated Tfh cells and IL-21 expression, and ameliorated mesangial matrix increase, moderate perivascular mononuclear cell infiltration, glomerular basement membrane thickening, and C3 immune complex deposition and other renal pathological features [15]. IL-6, p-STAT3, IFN-γ, IL-21 and Tfh cells levels were significantly elevated in SLE model mice. Artesunate is a semisynthetic derivative of artemisinin with anti-inflammatory and immunosuppressive effects. After artesunate intervention, IL-6, p-STAT3, IFN-γ, IL-21 and Tfh cells levels were significantly reduced and artesunate inhibited STAT3 signaling in a dose-dependent manner [16]. The up-regulation of Tfh cells differentiation mediated by abnormal IL-6/STAT3 signaling pathway is involved in the pathological process of AID. Inhibition of the IL-6/STAT3 signaling pathway can down-regulate Tfh cells differentiation and thus improve the state of AID.

IL-12 is also involved in the early differentiation phase of Tfh cells. IL-12 secreted by DCs, which are activated by TLRs or CD40L, induced the differentiation of naïve CD4^+^ T cells into Tfh cells via STAT4 in a dose-dependent manner. After IL-12 inhibition, Tfh cells differentiation was also inhibited, IL-21 and IFN-γ expression was reduced, and the ability of B cells to produce antibodies was correspondingly diminished [17,18].

The Non-obese diabetic (NOD) mice displayed similar lymphocyte alterations to Sjögren's syndrome (SS) patients. Using IL-12 antibody to intervene in the NOD mice can reduce the serum IL-12 level and the number of Tfh cells. Mesenchymal stem cell transplantation has also been found a similar phenomenon in the treatment of SS patients. Targeted regulation of IL-12 may be a new idea for SS treatment [19]. Hydroxychloroquine (HCQ) is a commonly used drug for the treatment of RA. HCQ downregulated the level of IL-12 in vitro experiment [20]. HCQ can inhibit the differentiation of Tfh cells induced by IL-12, and improve the incidence rate and score of arthritis in model mice [21]. The DCs level increased in RA patients, and HCQ further interfered with DC maturation by blocking TLR9 signal transduction [20]. The above research results suggest that the mechanism of HCQ in the treatment of RA may include inhibiting DCs and IL-12 secretion, and down-regulating the expression of Tfh cells, thereby improving the symptoms of RA.

In summary, DCs can act on the early differentiation of Tfh cells and participate in the disease process through signaling pathways such as IL-6/STAT3 or IL-12/STAT4. Biological agents play an important role in the treatment of AID, and tocilizumab, a monoclonal antibody targeting IL-6, has been approved for the treatment of RA [22]. However, there is a lack of clinical studies on the inhibitory effect of tocilizumab on Tfh cells, and the biologics targeting IL-12 have not been applied in the treatment of AID. Targeting the regulation of the early differentiation stage of Tfh cells in which DCs are involved has a promising future in the treatment of AID.

### Bcl-6 is a core transcription factor for the early differentiation of Tfh cells

The expression of Bcl-6 in Tfh cells was significantly higher than that in other Th cell subsets. Bcl-6 deficient mice cannot form GC and Bcl-6 deficient T cells cannot develop into Tfh cells [2]. Bcl-6 affects the level of Tfh cells in a gene dose-dependent manner. Blimp-1 and Bcl-6 antagonize each other and jointly regulate the differentiation of Th cell subsets [2]. Bcl-6 is regulated by factors such as STAT signals and affects Tfh cells differentiation through related miRNAs and cytokines.

The STAT signaling family regulates Bcl-6 and is closely related to the early differentiation of Tfh cells. IL-12 promoted the differentiation of Tfh-Th1-like cells by binding STAT1 and STAT4 on Bcl-6 and T-Box protein 21 (TBX21) genes. Silence the *STAT1* or *STAT4* could reduce the expression of Bcl-6, CXCR5, CXCR3 and T-box expressed in T cells (T-bet), and inhibit IL-12 mediated Tfh-Th1-like cells differentiation [23]. The regulation effect of the STAT3 signaling pathway on Bcl-6 is complex. STAT3 promoted Tfh cells differentiation by inducing Bcl-6 and inhibiting Blimp-1 expression [24]. In mouse CD4^+^ T cells, leptin activated the STAT3 and mammalian target of rapamycin (mTOR) pathways, upregulated the Bcl-6 expression, and enhanced the Tfh cells differentiation and function [25]. However, another study showed that STAT3 inhibited the activity of Bcl-6, and proposed that the inhibition or activation of Bcl-6 by STAT3 depends on the activation of other STAT signals and cytokines [26]. In the presence of IFN-I, both STAT5 and STAT3 can bind to *BCL-6* loci, and STAT5 has a strong binding force. STAT5 includes STAT5A and STAT5B. STAT5B inhibits Tfh cells through the Blimp-1/Bcl-6 axis. STAT5B deficiency can impair the generation of regulatory T (Treg) and follicular regulatory T (Tfr) cells, enhance the differentiation of naïve CD4^+^T cells into memory cells, and thus lead to the expansion and persistence of the circulating Tfh (cTfh) cells population [27]. Both phosphorylated STAT1 and STAT5 bound to the *BCL-6* site. IL-2 induced high levels of STAT5 phosphorylation, and pSTAT5 binding to the *BCL-6* gene locus can reduce pSTAT1 levels, thus inhibiting Tfh cells differentiation [28]. STAT5 also upregulated the antagonistic transcription factor Blimp-1 of Bcl-6, promoted Th1 cell differentiation, and inhibit Tfh cells differentiation [24]. Erythropoietin can also directly prevent Tfh cells differentiation and the production of GC B cells and autoantibodies in a STAT5-dependent manner, and the inhibiting effect of erythropoietin on Tfh cells may be related to the decreased expression of Bcl-6 [29]. In conclusion, in terms of affecting the expression of Bcl-6, STAT1 and STAT4 promote the early differentiation of Tfh cells, STAT5 inhibits the early differentiation of Tfh cells, and STAT3 plays a complex regulatory role.

The extracellular signal-regulated kinase (ERK) pathway is involved in Tfh cells differentiation, and inhibition of ERK2 promoted Tfh cells development. Zinc Finger Protein 831 (Zfp831) is a functional molecule downstream of CD28 and ICOS-ERK axes, and its expression is inhibited by ERK. Zfp831 promoted Tfh cells differentiation by directly up-regulating the expression of transcription factors Bcl-6. It was revealed that Zfp831 is bound to the *BCL-6* promoter and enhanced its expression, thus promoting Tfh cells differentiation [30]. Overexpression of Tox2 led to a substantial increase in *BCL-6* mRNA and protein expression, while Bcl-6 directly drove Tox2 expression during Tfh cells development. Clustering analysis revealed that IL-6 signaling and Tox2 together induced *BCL-6* gene expression [11]. IFN-γ promoted CD4^+^ T cell proliferation, enhanced Bcl-6 expression, and promote pre-Tfh cells differentiated to Tfh cells. While inhibition of IFN-γ expression, Tfh cells and autoantibody levels decreased. IFN-γR signal did not affect Bcl-6 expression in GC B cells [31]. Guanine nucleotide-binding protein subunit α13 (Gα13) signaling in T cells also decreased Bcl-6 and CXCR5 expression, thereby affecting Tfh cells differentiation [32]. In resting CD4^+^T cells, protein kinase D2 (Prkd2) restricted the entry of Bcl-6 into the nucleus, thereby inhibiting Tfh cells differentiation. Following immunization, Bcl-6 was up-regulated in CD4^+^ T cells, overcoming the inhibitory effect of Prkd2 and entering the nucleus to drive Tfh cells differentiation [33]. IκBNS belongs to the nuclear factor κB (NF-κB) repressor proteins. IκBNS promoted Bcl-6 expression by binding to the promoter region of *BCL-6*, thereby promoting Tfh cells differentiation [34]. The methyltransferase nuclear receptor binding SET domain protein 2 (Nsd2) -mediated modification of histone H3 lysine 36 dimethylation (*H3K36me2*) is required for Bcl-6 expression and Tfh cells differentiation. Lack of Nsd2 in T cells resulted in decreased Bcl-6 expression, impaired Tfh cells production and GC response, while Nsd2 overexpression increased Bcl-6 expression, and promoted Tfh cells differentiation and GC response [35].

Regulation of Bcl-6 expression may be used in the treatment of autoimmune diseases. After the intervention of TLR7 agonist imiquimod, SLE model mice and Tfh cells significantly decreased *BCL-6* mRNA expression and upregulated *PRDM1* (encoding Blimp-1) and *STAT5b* mRNA expression, and the number of Tfh cells and GC B cells were significantly reduced, while the anti-double-stranded DNA (anti-dsDNA) antibody and antinuclear antibody titers in serum were also significantly decreased [36]. Cyanidin is a natural pigment that can significantly down-regulate the activation of STAT3 and the expression of Bcl-6, increase the phosphorylation level of STAT5, and thus inhibit inflammation and joint destruction, as well as IL-21 and immunoglobulin G (IgG) production in adjuvant induced arthritic rats. *In vitro*, the expression of Rho associated coiled-coil forming protein kinase-2 (ROCK-2) in cells treated with cyanidin was also reduced, indicating that ROCK-2 is involved in controlling STATs phosphorylation and Bcl-6 expression to control T-cell differentiation [37]. The artemisinin analogue SM934 can alleviate the severity of arthritis in CIA mice, reduce bone erosion, reduce the levels of Tfh cells and Th17 cells, and inhibit the production of pathogenic antibodies. In vitro, it has been found that the therapeutic effect of SM934 may be related to inhibiting the expression of Bcl-6, thereby reducing the differentiation level of Tfh cells [38]. According to the study on ulcerative colitis model mice, curcumin inhibited the expression of Tfh cells related transcription factors Bcl-6 and p-STAT3, and significantly increased the protein levels of Blimp-1 and STAT3 in colon tissue, so as to achieve a therapeutic effect [39].

The expression of Bcl-6 is influenced by various signal transduction pathways and cytokines, specifically participate in the early differentiation of Tfh cells, and designating the Tfh cells lineage typing. Intervention in the above-mentioned signal transduction pathways and corresponding cytokines can regulate the expression of Bcl-6 and the differentiation of Tfh cells, which is of great significance for the treatment of immune related diseases.

### Ascl2 is another key transcription factor for the early differentiation of Tfh cells

Ascl2 is highly expressed in Tfh cells, and its expression may precede that of Bcl-6. The high expression state of Ascl2 increased *CXCR5* mRNA expression by approximately 60 times but did not affect the expression of *BCL-6*, *ICOS*, and *IL-21*. At the same time, it down-regulated C-C chemokine receptor 7 (CCR7) and IL-2 in T cells, accelerating T-cell migration to lymphatic follicles and differentiation of Tfh cells. Genome-wide analysis showed that Ascl2 directly regulated Tfh cells related genes and inhibited the expression of Th1 and Th17 characteristic genes. Knocking out the Ascl2 gene can lead to absolute damage to Tfh cells development [40]. After genetic mutations in Ascl2, the production of CXCR5 in CD4^+^ T cells was significantly reduced, and Ascl2 could also up-regulate the expression of IκBNS levels in CD4^+^ T cells to affect Tfh cells differentiation [34]. Ascl2 acts on the early stage of Tfh cells differentiation and may be a key transcription factor for Tfh cells early differentiation. In the SS mice model, it was found that the Ascl2 mRNA levels in model mice were significantly higher than those in control mice, which promoted the differentiation of Tfh cells in model mice and thus affected the autoimmune response of the SS model [41].

The naïve T cells differentiate toward Tfh cells in the T-cell region. DCs, Bcl-6, and Ascl2 play essential roles in this course. Various signaling pathways, cells, and cytokines are also involved in this process. Physiologically, the differentiation and maturation of Tfh cells are beneficial for the body to produce protective antibodies. Pathologically, excessive differentiation of Tfh cells can promote the expression of pathogenic antibodies in the body. The intervention of pathological early differentiation of Tfh cells by regulating signaling pathways, cell and cytokine levels is of great significance for disease treatment.

## Tfh cells migration and maturation stage

The second stage of Tfh cells differentiation occurs mainly in the T–B cell border and follicles of lymphoid tissue. Pre-Tfh cells are influenced by ICOS and PD-1 to migrate to the T–B cell border and interact with B cells. Activated B cells contribute to GC formation, and pre-Tfh cells further differentiate into mature Tfh cells within the GC, entering the final stage of Tfh cells differentiation. Relative to pre-Tfh cells, mature Tfh cells have increased expression of Bcl-6, CXCR5 and ICOS, which are involved in auxiliary B cell differentiation into mature plasma cells and antibody production. Tfh cells also have a secretory function, secreting factors such as IL-4, IL-10, IL-21 and IFN-γ.

### Bcl-6 promotes T–B cell interactions

In addition to influencing the early differentiation of Tfh cells, Bcl-6 also promotes T–B cell interactions. It was found that an intact *BCL-6* allele was sufficient to maintain long-term contact between activated T cells and cognate B cells. Enhanced T- and B-cell contact time was associated with enhanced calcium signaling, which mediated CD40L externalization from pre-Tfh cells, while T- and B-cell interactions depended on the delivery of CD40L from pre-Tfh cells to cognate B cells. Although T cells with insufficient Bcl-6 expression could express CD40L, they could not effectively deliver CD40L during brief contacts with cognate B cells, which might be associated with impaired calcium signaling pathways in T cells when Bcl-6 expression was impaired, and insufficient Bcl-6 expression in T cells also led to impaired CD40L signaling pathways in B cells [42].

### ICOS affects the differentiation and migration of Tfh cells and maintains their phenotype

ICOS externalizes CD40L in pre-Tfh cells by enhancing TCR dependent calcium signaling and binds to CD40 on the surface of B cells. At the same time, ICOS on the surface of Tfh cells also binds to ICOS ligand (ICOSL) on the surface of B cells [43]. The combination of ICOS and ICOSL can enhance the activation of phosphatidylinositol 3-kinase (PI3K), promoting Tfh cells to migrate across the T–B boundary into follicles. Further research has found that ICOS relies on its transmembrane domain to promote binding to tyrosine kinases lymphocyte cellspecific protein tyrosine kinase (LCK), thereby enhancing calcium signaling and activating PI3K [44]. ICOS promotes the formation of coordinated pseudopodia in T cells and promotes their persistent movement in a PI3K-dependent manner, thereby promoting T-cell recruitment from the T-B boundary to the follicles [45]. During the interaction between T and B cells, T cells secrete helper cytokines such as IL-4 and IL-21. Existing studies have shown that ICOS activates PI3K signaling through its tyrosine-based signaling motif, while demonstrating that the ICOS-PI3K signaling pathway plays a dominant role in increasing the expression of IL-21, IL-4, and promoting Tfh cells development [46]. Compared with Bcl-6, the upregulation of CXCR5 expression is more dependent on ICOS stimulation [47]. CXCR5 signal can also activate PI3K. ICOS deficient T cells cannot normally express CXCR5 and do not affect the expression of Bcl-6 and Ascl2, so ICOS can directly promote T-cell migration to follicles through the CXCR5 signal pathway [48]. Krüppel-like transcription factor 2 (Klf2) is an inhibitor of CXCR5, an activator of CCR7, and a transcription factor inhibiting Tfh cells differentiation. Research has shown that ICOS inhibits Klf2 through Forkhead box O1 (Foxo1) to maintain the Tfh cells phenotype. After blocking the ICOS signal of the fully developed Tfh cells, Tfh cells transformed into other Th subsets and migrates back to the T cell zone, resulting in the disappearance of subsequent GC reactions [49]. B cells expressing ICOSL are also crucial for optimal collaboration between antigen-specific T cells and B cells, promoting the production of normal Tfh cells and GC [45]. OX40 is coexpressed with ICOS on Tfh cells in and around GC. The interaction of OX40-OX40L and ICOS-ICOSL is critical to the later development of Tfh cells and the maintenance of GC B cells [50]. In SS patients, Tfh cells are enriched in salivary glands with GC and express ICOS. Blocking the expression of ICOS in vitro can effectively reduce the expression of IL-21, TNF-α, IL-6 and IL-8 [51]. In parasitic infectious diseases, ICOS is also necessary to produce high affinity protective antibodies [52]. It is demonstrated that ICOS is crucial for the differentiation and maintenance of Tfh cells, the formation of functioning GC, and the production of high affinity antibodies.

ICOS expression is influenced by a variety of signaling pathways, cytokines and immune cells. Tfh cells stimulated by CD3 and ICOS signals enhance the activity of mTOR complex2. Lack of mTOR complex2 signal leads to a decrease in ICOS expression, and impaired differentiation of the Tfh cells [53]. CD155^+^ L cell significantly promoted the proliferation of GC-Tfh, pre-Tfh and naïve CD4^+^T cells, confirming that CD155 signaling acts as a synergistic stimulus in all tonsillar CD4^+^ T cell subsets. It was found that CD155 signaling, affected pre-Tfh cells proliferation by promoting IL-10, IL-21, ICOS and soluble CD40 ligand (sCD40L) expression. In turn, these expression upregulation was also dependent on CD226 signaling in naive and pre-Tfh cells [54]. Thus, the CD155-CD226 axis drives the early to mid-stage of Tfh cells differentiation. IL-6 signaling is also necessary for the presence of ICOS expression in Tfh cells [55]. IL-6 expression increased after co-culture of fibroblast-like synoviocytes and anti-CD3/CD28-stimulated peripheral blood mononuclear cell (PBMC) from RA patients, which promotes the ratios of CD4^+^CXCR5^+^ICOS^+^cells [56]. The lack of E3 ubiquitin ligase Pellino1 (Peli1) promoted ICOS expression, and Peli1 mRNA expression was negatively correlated with ICOS expression on CD4^+^ T cells. It was revealed that enhanced ICOS expression in Peli1-deficient CD4^+^ T cells promoted Tfh cells differentiation [57]. Tfr cells also express ICOS and more than Tfh cells, suggesting that Tfr cells competitively inhibit pre-Tfh cells from binding to ICOSL [58]. MR2-1 is an anti-TNF-R2 antibody. ICOS and PD-1 expressed in MR2-1-stimulated Tfr cells, and Tfh cells differentiation was significantly inhibited [59]. The special AT-rich sequence-binding protein-1 (*SATB1*) gene repressed the *ICOS* promoter, and silencing *SATB1* in CD4^+^T cells can derepress ICOS and impair Tfr cell differentiation, thus promoting the formation of antigen-specific Tfh cells and driving isotype-switching antibody response [60]. E3 ubiquitin ligase Von Hippel-Lindau (VHL) deficiency leads to enhanced hypoxia-inducible factor 1α (HIF-1α)-mediated glycolytic activity, and it ultimately leads to reduced ICOS expression and thus inhibits Tfh cells differentiation [61]. The results suggest that the VHL-HIF-1α axis plays a major role in Tfh cells development through glycolytic-epigenetic reprogramming. ICOS signaling is required for induction and maintenance of the CIA model, inhibition of glycolysis may ameliorate arthritis of CIA mice [62]. ICOS-mediated T cell glycolytic pathway could be a potential therapeutic target for RA.

Some medicines could affect Tfh cells differentiation by inhibiting ICOS expression. Berberine downregulates the expression of ICOS through calcium signaling and inhibits the differentiation of pre-Tfh cells into Tfh cells [63]. Abatacept can also inhibit Tfh cells differentiation by reducing ICOS expression [64]. Oral administration of high doses of cyclosporine A (10 mg/Kg/d) in rats resulted in reduced expression of IL-6, IL-21, PD-1 and ICOS, indicating that T-cell activation was inhibited and that cyclosporine A could inhibit Tfh cells differentiation through ICOS [65].

ICOS is essential for the targeted migration of Tfh cells, late Tfh cells development, maintenance of Tfh cells phenotype and GC B-cell maintenance. Multiple cytokines, transcription factors, Tfr cells and medicines can affect Tfh cells differentiation by acting on the ICOS/ICOSL signaling pathway.

### PD-1 affects proliferation and function of the Tfh cells

PD-1-related research is currently a hot topic in oncology, which inhibits Tfh cells proliferation but positively regulates Tfh cells function and is an important marker of GC Tfh cells, while multiple cells, cytokines and signals affect Tfh cells differentiation by acting PD-1/PD-L1. In metastatic non-small cell lung cancer, It was demonstrated that regions with viable cancer cells were enriched for exhausted CD8^+^T cells, Treg cells, and Tfh cells, consistent with a pan-cancer analysis [66,67]. This recruitment was related to the capacity of transforming growth factor β (TGF-β) to drive chemokine C-X-C motif ligand 13 (CXCL13) expression, a chemoattractant of Tfh cells, by intratumor CD8^+^T cells [68]. And intratumoral ICOS^+^PD-1^+^CD4^+^Tfh cells preferentially recognize tumor-derived neoantigens compared with other CD4^+^ subsets [69]. Tfh cells exert an antitumor immune effect in a CD8^+^-dependent manner. The presence of Tfh cells is required for efficacy of anti-programmed cell death ligand-1 (anti-PD-L1) therapy. In patients treated with anti-PD-1 mAb, accumulation of Tfh cells and CD8^+^ at the tumor site is associated with outcome [68].

PD-1 is expressed at low levels on naïve CD4^+^ T cells, but a unique marker of GC Tfh cells that inhibits the activation of PI3K and migration of follicular T cells into the follicle. The inhibitory effect of PD-1 is mediated by PD-L1 expressed by bystander B cells [70]. As previously shown, CXCR5 is up-regulated during the early stages of Tfh cells differentiation, and CXCR5 activates PI3K to drive T-cell migration into the follicle. ICOS/ICOSL signaling is required to activate PI3K when Tfh cells cross the T-B border to the follicle, and PD-1 ensures that T cells high in ICOS expression enter the next stage of maturation [71]. PD-1 consistently suppresses ICOS-induced Tfh cells numbers by limiting the down-regulation of Klf2 in the GC response during TCR co-stimulation with ICOS [72]. Tfh cells differentiation also requires migratory DC that transport antigen to the lymph node, and migratory cDC2 uniquely carries antigen to the T-B border of the lymph node where Tfh cells initiation occurs. Migrating CD11b^+^cDC2s express the appropriate chemotactic receptors to homing to the T-B border and induce Tfh cells-dependent antibody response [73]. PD-1/PD-L1 interactions can inhibit Tfh cells differentiation. Studies have shown that loss of DC cell surface-specific PD-L1 results in a higher percentage of Tfh cells in the blood. Before entering B-cell follicles, PD-L1 on the surface of DC cells binds to pre-Tfh cells and controls Tfh cells differentiation and maintenance [74].

PD-1 deficiency significantly increased serum levels of monocyte chemoattractant protein-1 (MCP-1), IFN-γ, and IL-10 in infection treatment vaccine (ITV)-immunized mice. These elevated cytokines promoted the expansion of Plasmodium-specific Tfh cells and GC B cells in ITV-immunized PD-1^−/−^ mice [75]. It was also observed that PD-1 signaling deficiency, although increasing the number of Tfh cells, impairs the function of Tfh cells by reducing the ability of important cytokines such as IL-4 and IL-21 [76]. PD-1 is involved in the optimal concentrations of IL-21 product by Tfh cells, while PD-1 and PD-L1 can concentrate the Tfh cells from the follicle into the GC, and ensure the high-affinity B cells binding to mature Tfh cells. CXCR3 is expressed by T cells after stimulation of T-cell receptors and is the receptor for CXCL9 and CXCL10. CXCR3 is highly expressed outside the follicle, and PD-1 concentrates Tfh cells from the follicle into the GC by inhibiting CXCR3 expression. PD-1 correlates with the affinity of antibodies. It was shown that after anti-PD-1 immunotherapy, Tfh cells proliferated but antibody affinity decreased [77].

The OX40L-OX40 axis is of major importance for IL-21^+^ Tfh cells differentiation. Conventional DC2 (cDC2) and cDC1 subsets express similar concentrations of ICOSL, PDL1, but significantly increased expression of OX40L in cDC2 compared to cDC1. Blocking OX40L reduces the frequency of ICOS^+^PD-1^+^ Tfh cells [78]. Exposure to the envelope glycoprotein gp120 induced a higher proportion of PD-1^+^ T cells and Tfh cells expressing PD-1 or ICOS but also resulted in poor B-cell repertoire development. The high proportion of memory B cells positively correlates with a lower proportion of PD-1^+^CD4^+^ cells [79]. Thus, PD-1 can be defined as an envelope glycoprotein-induced inhibitory receptor that attenuates and/or delays the recall antibody responses by negatively acting on Tfh/B cells interactions.

PD-1 abnormality is common in patients with autoimmune diseases, and regulation of PD-1 expression can be used to treat these diseases. The cell phenotype of elevated Tfh cells in SLE patients was mainly TCF1^−^ and TCF1^+^ for the Treg cells. CD62L, TCF1 and PD-1 jointly promote the production of IL-21 and participate in the physiological and pathological process of SLE [80]. The level of Tfh cells in the spleen of CIA mice increased, and ICOS and PD-1 expression were up-regulated. After anti-TNFα and anti-IL-1β treated, the level of PD-1 and ICOS expression returned to normal and the number of Tfh cells decreased [81]. This provides a theoretical basis for the use of TNFα inhibitors in RA. Ethanol and acetate prevent arthritis by decreasing PD-1 expression in Tfh cells and inhibiting IL-21 secretion and formation of Tfh: B-cell conjugates [82].

ICOS participates in Tfh cells differentiation through calcium signaling, PI3K, Klf2, and OX40 promote T–B cell interactions, maintain Tfh cells phenotype, and promote GC formation. PD-1 safeguards Tfh cells differentiation and function. Meanwhile, various cytokines, cells and signals act on ICOS and PD-1 to participate in Tfh cells differentiation, which eventually lead to various physiopathological processes and cure or cause diseases.

### B cell–Tfh cell interactions

Pre-Tfh cells enter the follicle to interact with B cells. During the GC response, B-cell antigen binding affinity was positively correlated with the expression of C-C class chemokines 22 (CCL22). Under the stimulation of CD40, B cells in the GC up-regulate chemokines such as CCL22 and CCL17, which stimulate CC chemokine receptor 4 (CCR4) on Tfh cells to attract multiple helper cells from a distance and increase the chance of Th cell–B cell interactions. In the absence of affinity messages delivered to Tfh cells from CCL22 and CCL17, B cells would remain in the GC, suggesting that the CCR4-CCL17/22 axis promotes frequent contact between antigen-specific T cells and B cells and facilitates optimal GC formation [83]. Tfh cells interact with DCs and B cells at the T-B boundary on a long-term basis, but interact transiently when in contact with GC B cells, facilitating their search for and preferential help to B cells expressing high levels of peptide–major histocompatibility complex II (pMHCII), as well as providing competition for other GC B cell. The increased size and duration of GC Tfh cells contact with B cells prolonged Ca^2+^ signaling and improved the quality of the GC Tfh cell response, promoting the expression of IL-4 and IL-21 double-positive Tfh cells [84].

The transcription factor CR6-interacting factor 1 (CRIF1) is a multifunctional protein expressed in the nucleus and cytoplasm that regulates cell cycle and growth by regulating the expression of nerve growth factor IB, androgen receptor, STAT3 and others, and is also essential for mitochondrial function. The deletion of CRIF1 in B cells impairs mitochondrial oxidative function, increases STAT3 phosphorylation levels, expresses more ICOSL and intercellular adhesion molecule (ICAM) related to T-B cell interaction, and up-regulates the expression of Tfh cells characteristic genes such as signaling lymphocytic activation molecule family member 5 (*SLAMF5*), *IFNG* and C-X-C chemokine receptor 3 (*CXCR3*), thereby increasing the frequency of Tfh cells [85]. B cells expressing PD-L1 and PD-L2 and T cells expressing PD-1 regulate the production of long-lived plasma cells and memory B cells through T-B cell interactions [76]. IFN-γ, driven by TLR7 and produced by T cells, can control gene transcription for immune cell survival, proliferation, metabolism and autoimmunity through multiple cellular pathways. In vitro, IFN-αR and IFN-γR expression on B cells was elevated after TLR7 stimulation; in vivo, after IFN-γ signaling deletion, the renal pathological progression, GC size, Tfh cells response and antibody formation in TLR7-induced SLE-prone mice were attenuated, indicating that IFN-γ promotes the development of autoreactive B cells and the development of SLE [86].

During this stage of differentiation, pre-Tfh cells interact with antigen-specific homologous B cells to promote Tfh cells differentiation maturity which promote GC formation and secretion of specific antibodies. If we can specifically intervene in Tfh cells differentiation at this stage, it is of great significance to solve the clinical problem of treating disease without compromising autoimmunity.

### IL-21 is produced by Tfh cells and regulates Tfh cells differentiation

IL-21 is mainly produced by Tfh cells. T-B cell interactions are critical for IL-21 production [87]. In the GC, Tfh cells interact with B cells via ICOS/ICOSL to release large amounts of IL-21, and blocking the ICOS signaling pathway significantly reduces IL-21 production. Sustained TCR signaling is important for IL-21 production, and disruption of antigen presentation is detrimental to IL-21 production. Blockade of the CD40/CD40L pathway impairs the development of IL-21^+^CD4^+^ T cells during lymphocytic choriomeningitis virus (LCMV) Cl13 infection, and the proportion of Tfh cells and GC B cells is significantly reduced, suggesting that CD40-CD40L interactions are a critical role in IL-21 production. When IL-21 was eliminated in CXCR5^+^CD4^+^ T cells, LCMV-specific total IgG was reduced by approximately 30%, indicating that IL-21 produced by CXCR5^+^CD4^+^ T cells contributes to antiviral humoral immunity. Studies have shown a 2-fold reduction in the proportion of virus-specific CD8^+^ T cells in mice in which IL-21 was eliminated [88]. IL-21 production by Tfh cells is essential to promote CD8^+^ effector T-cell (Teff) differentiation, limit T-cell depletion and promote viral control. IL-21 is also important for maintaining Tfh cells homeostasis, promoting Tfh cells differentiation and inducing their self-expression in an autocrine manner [89]. αIL-21R, a monoclonal antibody to the IL-21 receptor, does not have cytotoxic effects on CD4^+^ T cells but can inhibit Tfh cells differentiation. IL-21 decreases Treg cell frequency and can be reversed by αIL-21R, indicating that IL-21 regulates Tfh/Tfr cells homeostasis and has significant implications for T- and B-cell differentiation and antibody production [90].

## Circulation Tfh cells classification and function

After interacting with B cells in the GC, Tfh cells have three outcomes: (i) reside in the primary GC, when CD90 expression of Tfh cells decreases; (ii) migrate to a new GC; (iii) down-regulate Bcl-6 to be converted into circulating memory Tfh cells, when memory Tfh cells express similar levels of Bcl-6 as naïve T cells [91–93]. Upon secondary antigen exposure, circulating memory Tfh cells are rapidly recruited to the GC after up-regulation of CXCR5 or Bcl-6, producing an effector antibody response.

### Tfh1, Tfh2, Tfh17 cells definition, function and influencing factors

Blood CD4^+^CXCR5^+^ T cells interact with naïve B cells to secrete IL-10 and IL-21 within 24 hours. CD4^+^CXCR5^−^ T cells hardly secrete IL-21 and secrete fewer CXCL13 chemokines than CD4^+^CXCR5^+^ T cells [94]. Blood CD4^+^CXCR5^+^ T cells with antigen specificity are circulating memory Tfh cells, which can be divided into three subpopulations based on differential expression of IFN-γ, IL-4, IL-17, CXCR3 and CCR6. Tfh1 cells can be defined as IFN-γ^+^CD4^+^CXCR5^+^ or CXCR3^+^CCR6^−^ T cells, and Tfh2 cells can be defined as IL-4^+^CD4^+^CXCR5^+^ or CXCR3^−^CCR6^−^ T cells, and Tfh17 cells can be defined as IL-17A^+^CD4^+^CXCR5^+^ or CXCR3^−^CCR6^+^ T cells [95].

When co-cultured with naïve B cells, only Tfh2 and Tfh17 cells produce IL-21 and immunoglobulins. Tfh2 and Tfh17 cells support chronic humoral immunity such as antibody production in autoimmune diseases, whereas Tfh1 cells support antibody responses in infection and vaccines with relatively short duration [96]. Activated circulating PD-1^+^CXCR5^+^ Tfh cells expanded in IgG4-related sclerosing cholangitis/autoimmune pancreatitis and PD-1 expression was enhanced in all Tfh cells subsets, correlating with disease activity and driving IgG4-committed B cell class switch and proliferation [97].

It was found that the T cell immunoglobulin and immune receptor tyrosine-based inhibitory motif domain (TIGIT) expression was higher in peripheral Tfh cells than in other Th cell subsets, TIGIT^+^ Tfh cells produced more IL-21 than TIGIT^−^ Tfh cells [98]. CD155 is a ligand for TIGIT. The activated cTfh cells express CD155, while memory B cells inhibit cTfh cells proliferation through the interaction of TIGIT on B cells and CD155 on cTfh cells [99]. Purinergic 2X7 (P2X7) promotes caspase-mediated Tfh cells pyroptosis and controls the development of pathogenic ICOS^+^CD4^+^ T cells secreting IFN-γ. Circulating Tfh cells in SLE patients are hyporesponsive to P2X7 stimulation and resistant to P2X7-mediated cytokine-driven proliferation inhibition [100]. Therefore, restoring P2X7 activity in SLE patients could help limit the increment of pathogenic autoantibodies and improve the patient's condition. IFN-α belongs to the type I interferon. It was found that after infection with foot-and-mouth disease virus (FMDV) recombinant adenovirus, IFN-α enhanced the production of memory Tfh cells subsets such as cTfh1 and cTfh2 cells with memory B cells, and also increased the expression level of Bcl-6 in memory Tfh cells. When the antigen attacked memory Tfh cells again, IFN-α enhanced the activation of memory Tfh cells and increased the expression levels of Bcl-6 and STAT1. It was hypothesized that IFN-α may enhance the differentiation of memory Tfh cells by regulating the transcription factors Bcl-6 and STAT1 [91]. Compared with healthy donors, the proportion of CXCR3^+^Tfh1 cells was significantly increased in PIK3CD Gain-of-function (GOF) patients, while the proportion of CCR6^+^Tfh17 cells and CXCR3^−^CCR6^−^cTfh cells was reduced, indicating that the overactive PI3K signaling pathway has a selective effect on the differentiation of cTfh cells that biases them toward the Th1 phenotype and away from the Th17 phenotype [101]. The frequency of cTfh2 and cTfh17 cells in CD4^+^ T cells of myasthenia gravis (MG) patients was significantly increased, and the levels of IL-21, IL-4, and IL-17a produced by ICOS^high^cTfh cells were significantly higher than those produced by ICOS^low^cTfh cells, indicating that high expression of ICOS in MG patients helps to exert the secretion function of Tfh2 and Tfh17 cells [102].

### ICOS, PD-1, and cTfh cells proliferation and function

In MG patients, cTfh cells increased with elevated ICOS expression, and ICOS^high^cTfh cells produced significantly higher levels of IL-21, IL-4, and IL-17a than ICOS^low^cTfh cells. The proportion of cTfh cells in CD4^+^T cells was correlated with disease severity. Immunotherapy may improve the abnormalities of cTfh cells [102]. Patients with MG combined with diabetes mellitus had increased cTfh cells and expressed ICOS at high levels, and activated cTfh cells were positively correlated with plasmablasts. Further studies showed that hyperglycemia promoted the differentiation and activation of Tfh cells, causing abnormal plasma cell differentiation and antibody secretion through the mTOR signaling pathway [103]. Tfh cells were increased and Tfr cells were decreased in the peripheral blood of RA patients. Tfr/Tfh cells ratio was negatively correlated with erythrocyte sedimentation rate, rheumatoid factor, hypersensitive C-reactive protein, IgG, DAS28 score, IL-21, CXCL13, and positively correlated with serum TGF-β level. Meanwhile, ICOS expression was higher in RA patients' blood and synovial membrane, suggesting that ICOS is also involved in RA pathogenesis [104]. Iguratimod (IGU) is an antirheumatic drug that has been found to act directly on B cells and inhibit antibody production in RA patients. It was found that IGU also further inhibited RA cTfh cells expression and function by inhibiting the mTOR/hypoxia-inducible factor (hif1α) /hexokinase 2 (hk2)/glucose metabolism axis, significantly reducing the expression of ICOS [105]. During the acute infection stage, human immunodeficiency virus type 1 (HIV-1) infection drives the expansion of cTfh cells, which in turn interferes with B-cell differentiation via the ICOS signaling pathway [106].

According to PD-1 expression, cTfh cells can be divided into resting and activated subsets, while cTfh cells with high PD-1 expression effectively promote antibody response [107]. In ovarian cancer patients, PD-1 was expressed more in Tfh cells compared to non-Tfh CD4^+^ cells. Moreover, PD-1^+^ Tfh cells secreted more IL-21 and IL-10 and promoted immunoglobulin secretion than PD-1^−^ Tfh cells [108]. Patients with multiple sclerosis exhibited significantly elevated PD1^+^cTfh cells frequencies; 36 months after autologous haematopoietic stem cell transplantation (AHSCT), PD1^+^cTfh cells frequencies were significantly reduced, consistent with normal subjects, suggesting that AHSCT can control the disease by modulating PD1^+^cTfh cells expression in patients with multiple sclerosis [109]. Patients with acute immune thrombotic thrombocytopenic purpura had decreased GC memory B cells, total cTfh cells, PD1^+^cTfh cells, and increased frequency of circulating plasma cells, while dysregulation of B cell and cTfh cells homeostasis may be caused by hyperactivation of T and B cells leading to differentiation of memory B cells to antibody-producing plasmablast and migration of circulating Tfh cells into the GC [110]. Peripheral blood PD-1^+^ICOS^+^Tfh cells in patients with primary SS (pSS) correlate strongly and positively with disease activity [111]. PD-1 expression in blood Tfh and Tfr cells of pSS patients was positively correlated with IL-21, and circulating CCR7^lo^PD-1^hi^Tfh cells suggest that patients are undergoing disease activity and glandular inflammation [112]. During the active phase of ulcerative colitis (UC), ICOS^+^, PD-1^+^ and ICOS^+^PD-1^+^ Tfh cells levels were significantly elevated, and significantly decreased upon reaching clinical remission; activated ICOS^+^PD-1^+^ Tfh cells were positively correlated with serum C-reactive protein (CRP) and Mayo scores, and ICOS^+^PD-1^+^ Tfh cells also correlated significantly with circulating new memory B cells, plasmablasts, and serum IgG, IL-4, and IL-21, suggesting that ICOS and PD-1 are involved in UC pathogenesis [113]. Mayo score is a criterion for evaluating the severity of UC patients’ conditions, with higher scores indicating greater severity. The expression levels of ICOS and PD-1, the corresponding ligands ICOSL and PD-L1 in B cells and their soluble forms (sICOS, sPD-1, sICOSL and sPDL-1) in the plasma of patients with MG revealed that ICOS/ICOSL and PD-1/PD-L1 are involved in the MG pathological process. sICOSL and sPD-1 expression abnormalities may interfere with the normal signaling of ICOS and PD-1 on Tfh cells, leading to Tfh cells overactivation and promoting disease progression [114]. Perinatally HIV-infected children have reduced peripheral Tfh cells levels and show high PD-1 expression, while the frequency of low-memory pTfh cells with this high PD-1 expression correlates with worsening disease status and activation of differentiated B-cell profiles, which may lead to an attenuated specific antibody response [115].

High expression of ICOS or PD-1 means that cTfh cells are in an activated state. Various cytokines, signals, cell surface markers, cells, etc. can affect cTfh cells expression through various pathways, thus worsening the disease state.

### Other functions of cTfh cells

6-Sulfo LacNAc monocytes (slanMo) is a subset of monocytes that have a greater ability to produce IL-12 and induce CD4^+^ T cell proliferation compared with DCs. Comparing slanMo cells and cTfh cells from SSc patients and HC, SlanMo cells were found to be increased in SSc patients and positively correlated with cTfh cells, speculating that SlanMo cells may be involved in the activation of cTfh cells in SSc patients [116]. After antigenic stimulation, cTfh cells express low or moderate levels of CD45RA. The number and function of antibodies decreased after vaccination in older adults compared to younger adults. After vaccination, cTfh cells was low in frequency despite an effector memory phenotype in older adults, while CCR7^+^CD45RA^+^cTfh cells were transiently increased in the blood of younger adults. Upon antigen reencounter, CCR7^+^CD45RA^+^cTfh cells showed higher levels of CD95, CD40L, CXCR3, and Bcl-6 [117]. After COVID-19 infection, older adults had higher levels of pro-inflammatory cytokines, more circulating plasmablasts, and a lower proportion of cTfh cells with antibodies, demonstrating that poor prognosis in older adults is associated with a pro-inflammatory state in *vivo* and an inability to generate appropriate antiviral response [118]. K/BxN mice is a kind of TCR transgenic autoimmune arthritis model, and anti-glucose-6-phosphate isomerase (gpi) autoantibodies serve as the disease index in the K/BxN model. The middle-aged K/BxN mice had greater ankle thickness and anti-gpi titers than young K/BxN mice; Tfh cells and GC responses significantly increased in the middle-aged group compared with the young group, regardless of the segmented filamentous bacteria status of induced Tfh cells; and it was demonstrated that the increased Tfh cells in middle-aged mice were due to effector Tfh cells accumulation rather than memory Tfh cells [119]. The above studies suggest that Tfh cells are also involved in the immune aging process.

It was also identified ‘Tfh13’ cells with an unusual cytokine profile (IL-13^hi^IL-4^hi^IL-5^hi^IL-21^lo^) co-expressing the transcription factors Bcl-6 and GATA3. Tfh13 cells are required for the production of high-affinity IgE and subsequent allergen-induced anaphylaxis. Blockade of Tfh13 cells may be an alternative therapeutic target to ameliorate allergic responses [120]. There is a super-functional Tfh-like cells subset in lupus-prone New Zealand Black x New Zealand White (NZBxW) mice, defined as IL-21^+^IFN-γ^high^PD-1^low^CD40L^high^CXCR5^−^Bcl-6^−^ T cells, which express high levels of TNF-α and IL-2 and help B cells to produce IgG in an IL-21 and CD40L-dependent manner [121].

cTfh cells are currently a hot research topic, and in the future there will be different defined subsets of cTfh cells, each representing its different functions and characteristics. In immune-related diseases, cTfh cells play an important role, and it is hoped that there will be more ideas for disease diagnosis and treatment with Tfh cells as the starting point in the future.

The occurrence and development of AID also involve other cells, including Treg cells. Treg cells are essential for immunosuppressive, maintenance self-tolerance and suppressing autoimmunity through the production of anti-inflammatory cytokines, and a deficiency of Treg cells results in fatal AID in humans and mice [122]. Increased plasticity of Tfh cells and CD4^+^T cells polyfunctionality with enriched memory Treg cell responses was demonstrated in RA patient synovial tissue, identified the presence of a novel population of pathogenic polyfunctional T cells that are enriched in the RA joint [123]. The administration of TGP had down-regulated the levels of Th1 cells and Th17 cells in CIA rats, and up-regulated the levels of Th2 cells and Treg cells. These results indicate that Treg can be AIDs' therapeutic target because of immunosuppressive functions [124].

## Conclusion

**Figure 1:**
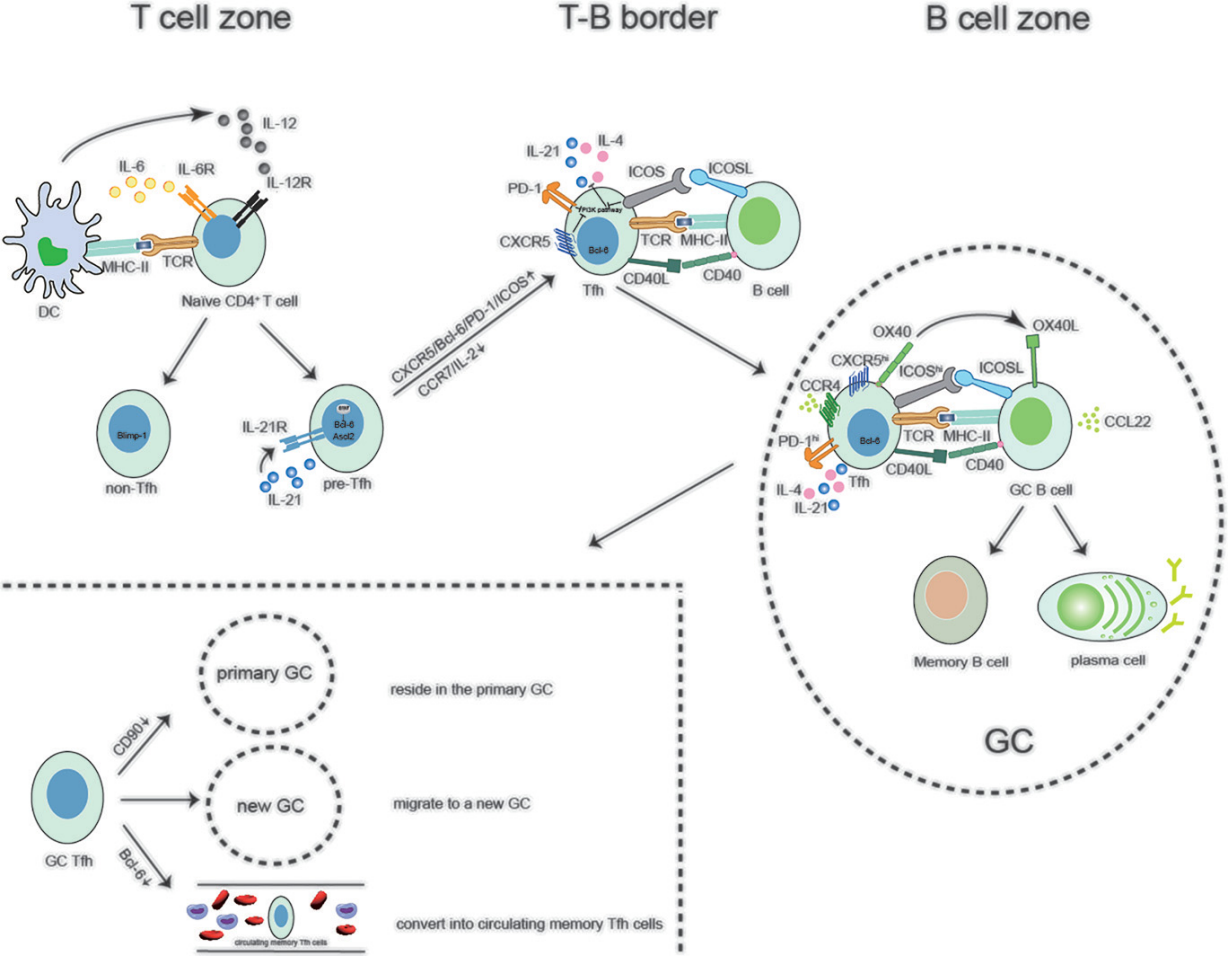
The differentiation courses of the Tfh cells Ascl2, achaete-scute homolog 2; Bcl-6, B-cell lymphoma-6; CCL22, C-C class chemokines 22; CCR7, C-C chemokine receptor 7; CD40L, CD40 ligand; CXCR5, chemokine receptor type 5; DC, dendritic cell; GC, germinal center; ICOS, inducible co-stimulator; IL-6, interleukin-6; IL-6R, IL-6 receptor α; MHC-II, major histocompatibility complex II; PD-1, programmed death-1; TCR, T-cell receptor; Tfh, follicular helper T cell.

Tfh cells have been the focus of research among T cells since their discovery in 2000. Numerous studies have demonstrated that Tfh cells’ differentiation requires the participation of cells and cytokines such as DCs, Bcl-6, Ascl2, ICOS, PD-1, and B cells, as well as the assistance of signaling pathways such as OX40/OX40L, IFNs, STATs, and Ascl2/IκBNS. When Tfh cells interact with B cells in the GC, they either stay in the GC or enter the blood to become memory Tfh cells (Figure 1). Since Tfh cells help B cells to produce antibodies and promote the formation of GC, they can be found in various immune-related diseases. However, the targeting of Tfh cells is also a challenge because Tfh cells can promote the production of pathogenic antibodies by B cells and also participate in the production of protective antibodies, which requires a lot of future research to balance the therapeutic and pathogenic functions of Tfh cells.

## Abbreviations

Tfh: Follicular helper T cell
Bcl-6: B-cell lymphoma-6
Th: helper T cell
CXCR5: C-X-C chemokine receptor 5
PD-1: programmed death-1
ICOS: inducible co-stimulator
CD40L: CD40 ligand
AID: autoimmune disease
DCs: dendritic cells
Ascl2: achaete-scute homologue 2
IL-6: Interleukin-6
STAT3: signal transducer and activator of transcription 3
RA: rheumatoid arthritis
DAS28: disease activity score in 28 joints
pSTAT3: phosphorylated STAT3
IFN-I: Type I interferon
TLRs: Toll-like Receptors
TCF-1: Transcription factor T cell factor 1
Tox2: Thymocyte selection-associated high mobility group box 2
Blimp-1: The B lymphocyte-inducible maturation protein 1
GC: germinal center
TGP: Total glucosides of paeony
CIA: collagen-induced arthritis
SLE: systemic lupus erythematosus
SS: Sjögren's syndrome
HCQ: Hydroxychloroquine
TBX21: T-Box protein 21
Tfr: follicular regulatory T
Treg: regulatory T
cTfh: circulating Tfh
ERK: The extracellular signal-regulated kinase
Zfp831: Zinc Finger Protein 831
Gα13: Guanine nucleotide-binding protein subunit α13
NF-κB: nuclear factor κB
CCR7: C-C chemokine receptor 7
PI3K: phosphatidylinositol 3-kinase
Klf2: Krüppel-like transcription factor 2
Foxo1: Forkhead box O1
PBMC: peripheral blood mononuclear cell
CXCL13: chemokine C-X-C motif ligand 13
MCP-1: monocyte chemoattractant protein-1
CCL22: C-C class chemokines 22
CCR4: CC chemokine receptor 4
GOF: Gain-of-function
MG: myasthenia gravis
gpi: glucose-6-phosphate isomerase

## References

[1] Schaerli P., Willimann K., Lang A.B., Lipp M., Loetscher P. and Moser B. (2000) CXC chemokine receptor 5 expression defines follicular homing T cells with B cell helper function. J. Exp. Med. 192, 1553–1562 10.1084/jem.192.11.1553 11104798 PMC2193097

[2] Yu D., Rao S., Tsai L.M., Lee S.K., He Y., Sutcliffe E.L. et al. (2009) The transcriptional repressor Bcl-6 directs T follicular helper cell lineage commitment. Immunity 31, 457–468 10.1016/j.immuni.2009.07.002 19631565

[3] Yin X., Chen S. and Eisenbarth S.C. (2021) Dendritic cell regulation of T helper cells. Annu. Rev. Immunol. 39, 759–790 10.1146/annurev-immunol-101819-025146 33710920

[4] Sakurai S., Furuhashi K., Horiguchi R., Nihashi F., Yasui H., Karayama M. et al. (2021) Conventional type 2 lung dendritic cells are potent inducers of follicular helper T cells in the asthmatic lung. Allergol Int. 70, 351–359 10.1016/j.alit.2021.01.008 33674189

[5] Deng J., Fan C., Gao X., Zeng Q., Guo R., Wei Y. et al. (2018) Signal transducer and activator of transcription 3 hyperactivation associates with follicular helper T cell differentiation and disease activity in rheumatoid arthritis. Front Immunol. 9, 1226 10.3389/fimmu.2018.01226 29915585 PMC5994589

[6] Ndeupen S., Bouteau A., Herbst C., Qin Z., Jacobsen S., Powers N.E. et al. (2022) Langerhans cells and cDC1s play redundant roles in mRNA-LNP induced protective anti-influenza and anti-SARS-CoV-2 immune responses. PLoS Pathog. 18, e1010255 10.1371/journal.ppat.1010255 35073387 PMC8812972

[7] Cucak H., Yrlid U., Reizis B., Kalinke U. and Johansson-Lindbom B. (2009) Type I interferon signaling in dendritic cells stimulates the development of lymph-node-resident T follicular helper cells. Immunity 31, 491–501 10.1016/j.immuni.2009.07.005 19733096

[8] Chakarov S. and Fazilleau N. (2014) Monocyte-derived dendritic cells promote T follicular helper cell differentiation. EMBO Mol Med. 6, 590–603 10.1002/emmm.201403841 24737871 PMC4023883

[9] Yao C., Zurawski S.M., Jarrett E.S., Chicoine B., Crabtree J., Peterson E.J. et al. (2015) Skin dendritic cells induce follicular helper T cells and protective humoral immune responses. J. Allergy Clin. Immunol. 136, 1387.e977–1397.e977 10.1016/j.jaci.2015.04.001 25962902 PMC4639468

[10] Choi Y.S., Gullicksrud J.A., Xing S., Zeng Z., Shan Q., Li F. et al. (2015) LEF-1 and TCF-1 orchestrate T(FH) differentiation by regulating differentiation circuits upstream of the transcriptional repressor Bcl6. Nat. Immunol. 16, 980–990 10.1038/ni.3226 26214741 PMC4545301

[11] Xu W., Zhao X., Wang X., Feng H., Gou M., Jin W. et al. (2019) The transcription factor Tox2 drives T follicular helper cell development via regulating chromatin accessibility. Immunity 51, 826.e5–839.e5 10.1016/j.immuni.2019.10.006 31732165

[12] Johnston R.J., Poholek A.C., DiToro D., Yusuf I., Eto D., Barnett B. et al. (2009) Bcl6 and Blimp-1 are reciprocal and antagonistic regulators of T follicular helper cell differentiation. Science 325, 1006–1010 10.1126/science.1175870 19608860 PMC2766560

[13] Kim S.J., Zou Y.R., Goldstein J., Reizis B. and Diamond B. (2011) Tolerogenic function of Blimp-1 in dendritic cells. J. Exp. Med. 208, 2193–2199 10.1084/jem.20110658 21948081 PMC3201204

[14] Li H., Cao X.Y., Dang W.Z., Jiang B., Zou J. and Shen X.Y. (2019) Total Glucosides of Paeony protects against collagen-induced mouse arthritis via inhibiting follicular helper T cell differentiation. Phytomedicine 65, 153091 10.1016/j.phymed.2019.153091 31654988

[15] Jang S.G., Lee J., Hong S.M., Song Y.S., Kim M.J., Kwok S.K. et al. (2021) Niclosamide suppresses the expansion of follicular helper T cells and alleviates disease severity in two murine models of lupus via STAT3. J Transl Med. 19, 86 10.1186/s12967-021-02760-2 33632240 PMC7908700

[16] Dang W.Z., Li H., Jiang B., Nandakumar K.S., Liu K.F., Liu L.X. et al. (2019) Therapeutic effects of artesunate on lupus-prone MRL/lpr mice are dependent on T follicular helper cell differentiation and activation of JAK2-STAT3 signaling pathway. Phytomedicine 62, 152965 10.1016/j.phymed.2019.152965 31129432

[17] Schmitt N., Morita R., Bourdery L., Bentebibel S.E., Zurawski S.M., Banchereau J. et al. (2009) Human dendritic cells induce the differentiation of interleukin-21-producing T follicular helper-like cells through interleukin-12. Immunity 31, 158–169 10.1016/j.immuni.2009.04.016 19592276 PMC2731623

[18] Martin-Gayo E., Gao C., Chen H.R., Ouyang Z., Kim D., Kolb K.E. et al. (2020) Immunological fingerprints of controllers developing neutralizing HIV-1 antibodies. Cell Rep. 30, 984.e4–996.e4 10.1016/j.celrep.2019.12.087 31995767 PMC6990401

[19] Shi B., Qi J., Yao G., Feng R., Zhang Z., Wang D. et al. (2018) Mesenchymal stem cell transplantation ameliorates Sjögren's syndrome via suppressing IL-12 production by dendritic cells. Stem Cell Res Ther. 9, 308 10.1186/s13287-018-1023-x 30409219 PMC6225717

[20] Han J., Li X., Luo X., He J., Huang X., Zhou Q. et al. (2020) The mechanisms of hydroxychloroquine in rheumatoid arthritis treatment: Inhibition of dendritic cell functions via Toll like receptor 9 signaling. Biomed. Pharmacother. 132, 110848 10.1016/j.biopha.2020.110848 33049581 PMC7547638

[21] Han J., Zhou Q., Li X., He J., Han Y., Jie H. et al. (2018) Novel function of hydroxychloroquine: down regulation of T follicular helper cells in collagen-induced arthritis. Biomed. Pharmacother. 97, 838–843 10.1016/j.biopha.2017.10.132 29136759

[22] Smolen J.S., Landewé R.B.M., Bijlsma J.W.J., Burmester G.R., Dougados M., Kerschbaumer A. et al. (2020) EULAR recommendations for the management of rheumatoid arthritis with synthetic and biological disease-modifying antirheumatic drugs: 2019 update. Ann. Rheum. Dis. 79, 685–699 10.1136/annrheumdis-2019-216655 31969328

[23] Ma X., Nakayamada S., Kubo S., Sakata K., Yamagata K., Miyazaki Y. et al. (2018) Expansion of T follicular helper-T helper 1 like cells through epigenetic regulation by signal transducer and activator of transcription factors. Ann. Rheum. Dis. 77, 1354–1361 10.1136/annrheumdis-2017-212652 29853448

[24] Ray J.P., Marshall H.D., Laidlaw B.J., Staron M.M., Kaech S.M. and Craft J. (2014) Transcription factor STAT3 and type I interferons are corepressive insulators for differentiation of follicular helper and T helper 1 cells. Immunity 40, 367–377 10.1016/j.immuni.2014.02.005 24631156 PMC3992517

[25] Deng J., Chen Q., Chen Z., Liang K., Gao X., Wang X. et al. (2021) The metabolic hormone leptin promotes the function of TFH cells and supports vaccine responses. Nat Commun. 12, 3073 10.1038/s41467-021-23220-x 34031386 PMC8144586

[26] Wu H., Xu L.L., Teuscher P., Liu H., Kaplan M.H. and Dent A.L. (2015) An inhibitory role for the transcription factor stat3 in controlling IL-4 and Bcl6 expression in follicular helper T cells. J. Immunol. 195, 2080–2089 10.4049/jimmunol.1500335 26188063 PMC4546859

[27] Pelham S.J., Caldirola M.S., Avery D.T., Mackie J., Rao G., Gothe F. et al. (2022) STAT5B restrains human B-cell differentiation to maintain humoral immune homeostasis. J. Allergy Clin. Immunol. 150, 931–946 10.1016/j.jaci.2022.04.011 35469842

[28] Jiang Q., Wang J., Jiang H., Li W., Sun Y., Shan Y. et al. (2022) Competitive binding of transcription factors underlies flexibility of T peripheral helper cells and T follicular helper cells in SLE. Rheumatology (Oxford). 61, 4547–4557 10.1093/rheumatology/keac112 35191465

[29] Guglielmo C., Bin S., Cantarelli C., Hartzell S., Angeletti A., Donadei C. et al. (2021) Erythropoietin reduces auto- and alloantibodies by inhibiting T follicular helper cell differentiation. J. Am. Soc. Nephrol. 32, 2542–2560 10.1681/ASN.2021010098 34261755 PMC8722788

[30] Wan S., Ni L., Zhao X., Liu X., Xu W., Jin W. et al. (2021) Costimulation molecules differentially regulate the ERK-Zfp831 axis to shape T follicular helper cell differentiation. Immunity 54, 2740.e6–2755.e6 10.1016/j.immuni.2021.09.018 34644536

[31] Lee S.K., Silva D.G., Martin J.L., Pratama A., Hu X., Chang P.P. et al. (2012) Interferon-γ excess leads to pathogenic accumulation of follicular helper T cells and germinal centers. Immunity 37, 880–892 10.1016/j.immuni.2012.10.010 23159227

[32] Kuen D.S., Park M., Ryu H., Choi G., Moon Y.H., Kim J.O. et al. (2021) Critical regulation of follicular helper T cell differentiation and function by Gα13 signaling. Proc Natl Acad Sci U.S.A. 118, e2108376118 10.1073/pnas.2108376118 34663730 PMC8639339

[33] Misawa T., SoRelle J.A., Choi J.H., Yue T., Wang K.W., McAlpine W. et al. (2020) Mutual inhibition between Prkd2 and Bcl6 controls T follicular helper cell differentiation. Sci Immunol. 5, eaaz0085 10.1126/sciimmunol.aaz0085 31980486 PMC7278039

[34] Hosokawa J., Suzuki K., Meguro K., Tanaka S., Maezawa Y., Suto A. et al. (2017) IκBNS enhances follicular helper T-cell differentiation and function downstream of ASCl2. J. Allergy Clin. Immunol. 140, 288.e8–291.e8 10.1016/j.jaci.2016.10.047 28040417

[35] Long X., Zhang L., Zhang Y., Min M., Lin B., Chen J. et al. (2020) Histone methyltransferase Nsd2 is required for follicular helper T cell differentiation. J. Exp. Med. 217, e20190832 10.1084/jem.20190832 31636135 PMC7037247

[36] Duan X., Shen C., Zhang X., Wu L., Chen J., Ma B. et al. (2020) Toll-like receptor 7 agonist imiquimod prevents the progression of SLE in MRL/lpr mice via inhibiting the differentiation of T follicular helper cells. Int. Immunopharmacol. 80, 106239 10.1016/j.intimp.2020.106239 32007709

[37] Samarpita S. and Rasool M. (2021) Cyanidin restores Th17/Treg balance and inhibits T follicular helper cell differentiation via modulation of ROCK2 signaling in an experimental model of rheumatoid arthritis. Int. Immunopharmacol. 101, 108359 10.1016/j.intimp.2021.108359 34863656

[38] Lin Z.M., Yang X.Q., Zhu F.H., He S.J., Tang W. and Zuo J.P. (2016) Artemisinin analogue SM934 attenuate collagen-induced arthritis by suppressing T follicular helper cells and T helper 17 cells. Sci. Rep. 6, 38115 10.1038/srep38115 27897259 PMC5126690

[39] Wang H.Y., Ge W., Liu S.Q., Long J., Jiang Q.Q., Zhou W. et al. (2022) Curcumin inhibits T follicular helper cell differentiation in mice with dextran sulfate sodium (DSS)-induced colitis. Am. J. Chin. Med. 50, 275–293 10.1142/S0192415X22500100 34931590

[40] Liu X., Chen X., Zhong B., Wang A., Wang X., Chu F. et al. (2014) Transcription factor achaete-scute homologue 2 initiates follicular T-helper-cell development. Nature 507, 513–518 10.1038/nature12910 24463518 PMC4012617

[41] Otsuka K., Yamada A., Saito M., Ushio A., Sato M., Kisoda S. et al. (2019) Achaete-scute homologue 2-regulated follicular helper T cells promote autoimmunity in a murine model for Sjögren syndrome. Am. J. Pathol. 189, 2414–2427 10.1016/j.ajpath.2019.08.008 31539517

[42] Liu D., Yan J., Sun J., Liu B., Ma W., Li Y. et al. (2021) BCL6 controls contact-dependent help delivery during follicular T-B cell interactions. Immunity 54, 2245.e4–2255.e4 10.1016/j.immuni.2021.08.003 34464595 PMC8528402

[43] Liu D., Xu H., Shih C., Wan Z., Ma X., Ma W. et al. (2015) T-B-cell entanglement and ICOSL-driven feed-forward regulation of germinal centre reaction. Nature 517, 214–218 10.1038/nature13803 25317561

[44] Wan Z., Shao X., Ji X., Dong L., Wei J., Xiong Z. et al. (2020) Transmembrane domain-mediated Lck association underlies bystander and costimulatory ICOS signaling. Cell Mol Immunol. 17, 143–152 10.1038/s41423-018-0183-z 30523347 PMC7000777

[45] Xu H., Li X., Liu D., Li J., Zhang X., Chen X. et al. (2013) Follicular T-helper cell recruitment governed by bystander B cells and ICOS-driven motility. Nature 496, 523–527 10.1038/nature12058 23619696

[46] Gigoux M., Shang J., Pak Y., Xu M., Choe J., Mak T.W. et al. (2009) Inducible costimulator promotes helper T-cell differentiation through phosphoinositide 3-kinase. Proc Natl Acad Sci U.S.A. 106, 20371–20376 10.1073/pnas.0911573106 19915142 PMC2787139

[47] Chen X., Ma W., Zhang T., Wu L. and Qi H. (2015) Phenotypic Tfh development promoted by CXCR5-controlled re-localization and IL-6 from radiation-resistant cells. Protein Cell. 6, 825–832 10.1007/s13238-015-0210-0 26404031 PMC4624673

[48] Wan Z., Lin Y., Zhao Y. and Qi H. (2019) TFH cells in bystander and cognate interactions with B cells. Immunol. Rev. 288, 28–36 10.1111/imr.12747 30874359

[49] Weber J.P., Fuhrmann F., Feist R.K., Lahmann A., Al Baz M.S., Gentz L.J. et al. (2015) ICOS maintains the T follicular helper cell phenotype by down-regulating Krüppel-like factor 2. J. Exp. Med. 212, 217–233 10.1084/jem.20141432 25646266 PMC4322049

[50] Tahiliani V., Hutchinson T.E., Abboud G., Croft M. and Salek-Ardakani S. (2017) OX40 cooperates with ICOS to amplify follicular Th cell development and germinal center reactions during infection. J. Immunol. 198, 218–228 10.4049/jimmunol.1601356 27895177 PMC5173420

[51] Pontarini E., Murray-Brown W.J., Croia C., Lucchesi D., Conway J., Rivellese F. et al. (2020) Unique expansion of IL-21+ Tfh and Tph cells under control of ICOS identifies Sjögren’s syndrome with ectopic germinal centres and MALT lymphoma. Ann. Rheum. Dis. 79, 1588–1599 10.1136/annrheumdis-2020-217646 32963045 PMC7677495

[52] Wikenheiser D.J., Ghosh D., Kennedy B. and Stumhofer J.S. (2016) The costimulatory molecule ICOS regulates host Th1 and follicular Th cell differentiation in response to Plasmodium chabaudi chabaudi AS infection. J. Immunol. 196, 778–791 10.4049/jimmunol.1403206 26667167 PMC4705592

[53] Hao Y., Wang Y., Liu X., Yang X., Wang P., Tian Q. et al. (2018) The Kinase Complex mTOR Complex 2 Promotes the Follicular Migration and Functional Maturation of Differentiated Follicular Helper CD4+ T Cells During Viral Infection. Front Immunol. 9, 1127 10.3389/fimmu.2018.01127 29875775 PMC5974104

[54] Yasutomi M., Christiaansen A.F., Imai N., Martin-Orozco N., Forst C.V., Chen G. et al. (2022) CD226 and TIGIT Cooperate in the Differentiation and Maturation of Human Tfh Cells. Front Immunol. 13, 840457 10.3389/fimmu.2022.840457 35273617 PMC8902812

[55] Wong K.A., Harker J.A., Dolgoter A., Marooki N. and Zuniga E.I. (2019) T Cell-Intrinsic IL-6R Signaling Is Required for Optimal ICOS Expression and Viral Control during Chronic Infection. J. Immunol. 203, 1509–1520 10.4049/jimmunol.1801567 31413107 PMC8131195

[56] Tang Y., Wang B., Sun X., Li H., Ouyang X., Wei J. et al. (2017) Rheumatoid arthritis fibroblast-like synoviocytes co-cultured with PBMC increased peripheral CD4+ CXCR5+ ICOS+ T cell numbers. Clin. Exp. Immunol. 190, 384–393 10.1111/cei.13025 28833034 PMC5680054

[57] Huang X., Hao S., Liu J., Huang Y., Liu M., Xiao C. et al. (2021) The ubiquitin ligase Peli1 inhibits ICOS and thereby Tfh-mediated immunity. Cell Mol Immunol. 18, 969–978 10.1038/s41423-021-00660-5 33707688 PMC8115645

[58] Noël G., Fontsa M.L., Garaud S., De Silva P., de Wind A., Van den Eynden G.G. et al. (2021) Functional Th1-oriented T follicular helper cells that infiltrate human breast cancer promote effective adaptive immunity. J. Clin. Invest. 131, e139905 10.1172/JCI139905 34411002 PMC8483751

[59] Kawano S., Mitoma H., Inokuchi S., Yamauchi Y., Yokoyama K., Nogami J. et al. (2022) TNFR2 Signaling Enhances Suppressive Abilities of Human Circulating T Follicular Regulatory Cells. J. Immunol. 208, 1057–1065 10.4049/jimmunol.2100323 35149531

[60] Chaurio R.A., Anadon C.M., Lee Costich T., Payne K.K., Biswas S., Harro C.M. et al. (2022) TGF-β-mediated silencing of genomic organizer SATB1 promotes Tfh cell differentiation and formation of intra-tumoral tertiary lymphoid structures. Immunity 55, 115.e9–128.e9 10.1016/j.immuni.2021.12.007 35021053 PMC8852221

[61] Zhu Y., Zhao Y., Zou L., Zhang D., Aki D. and Liu Y.C. (2019) The E3 ligase VHL promotes follicular helper T cell differentiation via glycolytic-epigenetic control. J. Exp. Med. 216, 1664–1681 10.1084/jem.20190337 31123085 PMC6605754

[62] Panneton V., Bagherzadeh Yazdchi S., Witalis M., Chang J. and Suh W.K. (2018) ICOS signaling controls induction and maintenance of collagen-induced arthritis. J. Immunol. 200, 3067–3076 10.4049/jimmunol.1701305 29581356

[63] Vita A.A. and Pullen N.A. (2022) Exploring the mechanism of berberine-mediated Tfh cell immunosuppression. Phytomedicine 105, 154343 10.1016/j.phymed.2022.154343 35901597 PMC9948547

[64] Glatigny S., Höllbacher B., Motley S.J., Tan C., Hundhausen C., Buckner J.H. et al. (2019) Abatacept Targets T Follicular Helper and Regulatory T Cells, Disrupting Molecular Pathways That Regulate Their Proliferation and Maintenance. J. Immunol. 202, 1373–1382 10.4049/jimmunol.1801425 30683697 PMC6481683

[65] Steines L., Poth H., Schuster A., Amann K., Banas B. and Bergler T. (2021) Disruption of Tfh:B Cell Interactions Prevents Antibody-Mediated Rejection in a Kidney Transplant Model in Rats: Impact of Calcineurin Inhibitor Dose. Front Immunol. 12, 657894 10.3389/fimmu.2021.657894 34135891 PMC8201497

[66] Pai J.A., Hellmann M.D., Sauter J.L., Mattar M., Rizvi H., Woo H.J. et al. (2023) Lineage tracing reveals clonal progenitors and long-term persistence of tumor-specific T cells during immune checkpoint blockade. Cancer Cell. 41, 776.e7–790.e7 10.1016/j.ccell.2023.03.009 37001526 PMC10563767

[67] Zheng L., Qin S., Si W., Wang A., Xing B., Gao R. et al. (2021) Pan-cancer single-cell landscape of tumor-infiltrating T cells. Science 374, abe6474 10.1126/science.abe6474 34914499

[68] Niogret J., Berger H., Rebe C., Mary R., Ballot E., Truntzer C. et al. (2021) Follicular helper-T cells restore CD8+-dependent antitumor immunity and anti-PD-L1/PD-1 efficacy. J Immunother Cancer 9, e002157 10.1136/jitc-2020-002157 34103351 PMC8190041

[69] Duhen R., Fesneau O., Samson K.A., Frye A.K., Beymer M., Rajamanickam V. et al. (2022) PD-1 and ICOS coexpression identifies tumor-reactive CD4+ T cells in human solid tumors. J. Clin. Invest. 132, e156821 10.1172/JCI156821 35439168 PMC9197519

[70] Jensen O., Trivedi S., Meier J.D., Fairfax K.C., Hale J.S. and Leung D.T. (2022) A subset of follicular helper-like MAIT cells can provide B cell help and support antibody production in the mucosa. Sci Immunol. 7, eabe8931 10.1126/sciimmunol.abe8931 35030034 PMC9001248

[71] Shi J., Hou S., Fang Q., Liu X., Liu X. and Qi H. (2018) PD-1 Controls Follicular T Helper Cell Positioning and Function. Immunity 49, 264.e4–274.e4 10.1016/j.immuni.2018.06.012 30076099 PMC6104813

[72] Mizuno R., Sugiura D., Shimizu K., Maruhashi T., Watada M., Okazaki I.M. et al. (2019) PD-1 Primarily Targets TCR Signal in the Inhibition of Functional T Cell Activation. Front Immunol. 10, 630 10.3389/fimmu.2019.00630 31001256 PMC6455061

[73] Krishnaswamy J.K., Gowthaman U., Zhang B., Mattsson J., Szeponik L., Liu D. et al. (2017) Migratory CD11b+ conventional dendritic cells induce T follicular helper cell-dependent antibody responses. Sci Immunol. 2, eaam9169 10.1126/sciimmunol.aam9169 29196450 PMC7847246

[74] Sage P.T., Schildberg F.A., Sobel R.A., Kuchroo V.K., Freeman G.J. and Sharpe A.H. (2018) Dendritic Cell PD-L1 Limits Autoimmunity and Follicular T Cell Differentiation and Function. J. Immunol. 200, 2592–2602 10.4049/jimmunol.1701231 29531164 PMC6054131

[75] Liu T., Cheng X., Ding Y., Zhu F., Fu Y., Peng X. et al. (2018) PD-1 deficiency promotes TFH cells expansion in ITV-immunized mice by upregulating cytokines secretion. Parasit Vectors 11, 397 10.1186/s13071-018-2984-4 29980219 PMC6035468

[76] Good-Jacobson K.L., Szumilas C.G., Chen L., Sharpe A.H., Tomayko M.M. and Shlomchik M.J. (2010) PD-1 regulates germinal center B cell survival and the formation and affinity of long-lived plasma cells. Nat. Immunol. 11, 535–542 10.1038/ni.1877 20453843 PMC2874069

[77] Herati R.S., Knorr D.A., Vella L.A., Silva L.V., Chilukuri L., Apostolidis S.A. et al. (2022) PD-1 directed immunotherapy alters Tfh and humoral immune responses to seasonal influenza vaccine. Nat. Immunol. 23, 1183–1192 10.1038/s41590-022-01274-3 35902637 PMC9880663

[78] Naessens T., Morias Y., Hamrud E., Gehrmann U., Budida R., Mattsson J. et al. (2020) Human Lung Conventional Dendritic Cells Orchestrate Lymphoid Neogenesis during Chronic Obstructive Pulmonary Disease. Am. J. Respir. Crit. Care Med. 202, 535–548 10.1164/rccm.201906-1123OC 32255375 PMC7616955

[79] Yuan L., Chen W.J., Wang J.Y., Li Y., Tian D., Wang M.X. et al. (2018) Divergent Primary Immune Responses Induced by Human Immunodeficiency Virus-1 gp120 and Hepatitis B Surface Antigen Determine Antibody Recall Responses. Virol Sin. 33, 502–514 10.1007/s12250-018-0074-6 30569292 PMC6335216

[80] Zeng X., Zheng M., Liu T., Bahabayi A., Kang R., Xu Q. et al. (2022) Changes in the expression of T-cell factor-1 in follicular helper T cells reflect the condition of systemic lupus erythematosus patients. Int. Immunopharmacol. 108, 108877 10.1016/j.intimp.2022.108877 35729844

[81] Zeng X., Lu S., Li M., Zheng M., Liu T., Kang R. et al. (2022) Inflammatory Cytokine-Neutralizing Antibody Treatment Prevented Increases in Follicular Helper T Cells and Follicular Regulatory T Cells in a Mouse Model of Arthritis. J Inflamm Res. 15, 3997–4011 10.2147/JIR.S355720 35860232 PMC9292064

[82] Azizov V., Dietel K., Steffen F., Dürholz K., Meidenbauer J., Lucas S. et al. (2020) Ethanol consumption inhibits TFH cell responses and the development of autoimmune arthritis. Nat Commun. 11, 1998 10.1038/s41467-020-15855-z 32332730 PMC7181688

[83] Liu B., Lin Y., Yan J., Yao J., Liu D., Ma W. et al. (2021) Affinity-coupled CCL22 promotes positive selection in germinal centres. Nature 592, 133–137 10.1038/s41586-021-03239-2 33597749

[84] Shulman Z., Gitlin A.D., Weinstein J.S., Lainez B., Esplugues E., Flavell R.A. et al. (2014) Dynamic signaling by T follicular helper cells during germinal center B cell selection. Science 345, 1058–1062 10.1126/science.1257861 25170154 PMC4519234

[85] Park J.S., Yang S., Hwang S.H., Choi J., Kwok S.K., Kong Y.Y. et al. (2022) B Cell-Specific Deletion of CR6-Interacting Factor 1 Drives Lupus-like Autoimmunity by Activation of Interleukin-17, Interleukin-6, and Pathogenic Follicular Helper T Cells in a Mouse Model. Arthritis Rheumatol. 74, 1211–1222 10.1002/art.42091 35166061

[86] Chodisetti S.B., Fike A.J., Domeier P.P., Singh H., Choi N.M., Corradetti C. et al. (2020) Type II but Not Type I IFN Signaling Is Indispensable for TLR7-Promoted Development of Autoreactive B Cells and Systemic Autoimmunity. J. Immunol. 204, 796–809 10.4049/jimmunol.1901175 31900342 PMC7002260

[87] Cui C., Wang J., Fagerberg E., Chen P.M., Connolly K.A., Damo M. et al. (2021) Neoantigen-driven B cell and CD4 T follicular helper cell collaboration promotes anti-tumor CD8 T cell responses. Cell 184, 6101.e13–6118.e13 10.1016/j.cell.2021.11.007 34852236 PMC8671355

[88] Zander R., Kasmani M.Y., Chen Y., Topchyan P., Shen J., Zheng S. et al. (2022) Tfh-cell-derived interleukin 21 sustains effector CD8+ T cell responses during chronic viral infection. Immunity 55, 475.e5–493.e5 10.1016/j.immuni.2022.01.018 35216666 PMC8916994

[89] Almanan M., Raynor J., Ogunsulire I., Malyshkina A., Mukherjee S., Hummel S.A. et al. (2020) IL-10-producing Tfh cells accumulate with age and link inflammation with age-related immune suppression. Sci Adv. 6, eabb0806 10.1126/sciadv.abb0806 32832688 PMC7439492

[90] Nian Y., Xiong Z., Zhan P., Wang Z., Xu Y., Wei J. et al. (2021) IL-21 Receptor Blockade Shifts the Follicular T Cell Balance and Reduces De Novo Donor-Specific Antibody Generation. Front Immunol. 12, 661580 10.3389/fimmu.2021.661580 33897706 PMC8064115

[91] Duan X., Sun P., Lan Y., Shen C., Zhang X., Hou S. et al. (2020) 1IFN-α Modulates Memory Tfh Cells and Memory B Cells in Mice, Following Recombinant FMDV Adenoviral Challenge. Front Immunol. 11, 701 10.3389/fimmu.2020.00701 32411135 PMC7200983

[92] Ma C.S., Wong N., Rao G., Avery D.T., Torpy J., Hambridge T. et al. (2015) Monogenic mutations differentially affect the quantity and quality of T follicular helper cells in patients with human primary immunodeficiencies. J. Allergy Clin. Immunol. 136, 993.e1–1006.e1 10.1016/j.jaci.2015.05.036 26162572 PMC5042203

[93] Yeh C.H., Finney J., Okada T., Kurosaki T. and Kelsoe G. (2022) Primary germinal center-resident T follicular helper cells are a physiologically distinct subset of CXCR5hiPD-1hi T follicular helper cells. Immunity 55, 272.e7–289.e7 10.1016/j.immuni.2021.12.015 35081372 PMC8842852

[94] Morita R., Schmitt N., Bentebibel S.E., Ranganathan R., Bourdery L., Zurawski G. et al. (2011) Human blood CXCR5(+)CD4(+) T cells are counterparts of T follicular cells and contain specific subsets that differentially support antibody secretion. Immunity 34, 108–121 10.1016/j.immuni.2010.12.012 21215658 PMC3046815

[95] Reinhardt R.L., Liang H.E. and Locksley R.M. (2009) Cytokine-secreting follicular T cells shape the antibody repertoire. Nat. Immunol. 10, 385–393 10.1038/ni.1715 19252490 PMC2714053

[96] Yao Y., Wang Z.Z., Huang A., Liu Y., Wang N., Wang Z.C. et al. (2022) TFH 2 cells associate with enhanced humoral immunity to SARS-CoV-2 inactivated vaccine in patients with allergic rhinitis. Clin Transl Med. 12, e717 10.1002/ctm2.717 35083875 PMC8792397

[97] Cargill T., Makuch M., Sadler R., Lighaam L.C., Peters R., van Ham M. et al. (2019) Activated T-Follicular Helper 2 Cells Are Associated With Disease Activity in IgG4-Related Sclerosing Cholangitis and Pancreatitis. Clin. Transl. Gastroenterol. 10, e00020 10.14309/ctg.0000000000000020 31033594 PMC6602789

[98] Akiyama M., Suzuki K., Yoshimoto K., Yasuoka H., Kaneko Y. and Takeuchi T. (2021) Peripheral TIGIT+ T Follicular Helper Cells That Produce High Levels of Interleukin-21 via OX40 Represent Disease Activity in IgG4-Related Disease. Front Immunol. 12, 651357 10.3389/fimmu.2021.651357 33936071 PMC8079782

[99] Asashima H., Axisa P.P., Pham T.H.G., Longbrake E.E., Ruff W.E., Lele N. et al. (2022) Impaired TIGIT expression on B cells drives circulating follicular helper T cell expansion in multiple sclerosis. J. Clin. Invest. 132, e156254 10.1172/JCI156254 36250467 PMC9566906

[100] Faliti C.E., Gualtierotti R., Rottoli E., Gerosa M., Perruzza L., Romagnani A. et al. (2019) P2X7 receptor restrains pathogenic Tfh cell generation in systemic lupus erythematosus. J. Exp. Med. 216, 317–336 10.1084/jem.20171976 30655308 PMC6363434

[101] Bier J., Rao G., Payne K., Brigden H., French E., Pelham S.J. et al. (2019) Activating mutations in PIK3CD disrupt the differentiation and function of human and murine CD4+ T cells. J. Allergy Clin. Immunol. 144, 236–253 10.1016/j.jaci.2019.01.033 30738173 PMC6612302

[102] Ashida S., Ochi H., Hamatani M., Fujii C., Kimura K., Okada Y. et al. (2021) Immune Skew of Circulating Follicular Helper T Cells Associates With Myasthenia Gravis Severity. Neurol Neuroimmunol Neuroinflamm 8, e945 10.1212/NXI.0000000000000945 33436376 PMC8105905

[103] Li T., Yang C.L., Du T., Zhang P., Zhou Y., Li X.L. et al. (2022) Diabetes mellitus aggravates humoral immune response in myasthenia gravis by promoting differentiation and activation of circulating Tfh cells. Clin. Immunol. 245, 109141 10.1016/j.clim.2022.109141 36270469

[104] Cao G., Wang P., Cui Z., Yue X., Chi S., Ma A. et al. (2020) An imbalance between blood CD4+CXCR5+Foxp3+ Tfr cells and CD4+CXCR5+Tfh cells may contribute to the immunopathogenesis of rheumatoid arthritis. Mol. Immunol. 125, 1–8 10.1016/j.molimm.2020.06.003 32610164

[105] Bai Z., Lu Z., Liu R., Tang Y., Ye X., Jin M. et al. (2022) Iguratimod Restrains Circulating Follicular Helper T Cell Function by Inhibiting Glucose Metabolism via Hif1α-HK2 Axis in Rheumatoid Arthritis. Front Immunol. 13, 757616 10.3389/fimmu.2022.757616 35720293 PMC9199372

[106] Lu X., Zhang X., Cheung A.K.L., Moog C., Xia H., Li Z. et al. (2022) Abnormal Shift in B Memory Cell Profile Is Associated With the Expansion of Circulating T Follicular Helper Cells via ICOS Signaling During Acute HIV-1 Infection. Front Immunol. 13, 837921 10.3389/fimmu.2022.837921 35222430 PMC8867039

[107] He J., Tsai L.M., Leong Y.A., Hu X., Ma C.S., Chevalier N. et al. (2013) Circulating precursor CCR7(lo)PD-1(hi) CXCR5^+^ CD4^+^ T cells indicate Tfh cell activity and promote antibody responses upon antigen reexposure. Immunity 39, 770–781 10.1016/j.immuni.2013.09.007 24138884

[108] Li L., Ma Y., Xu Y. and Maerkeya K. (2018) TIM-3 expression identifies a distinctive PD-1+ follicular helper T cell subset, with reduced interleukin 21 production and B cell help function in ovarian cancer patients. Int. Immunopharmacol. 57, 139–146 10.1016/j.intimp.2018.02.016 29482158

[109] Visweswaran M., Hendrawan K., Massey J.C., Khoo M.L., Ford C.D., Zaunders J.J. et al. (2022) Sustained immunotolerance in multiple sclerosis after stem cell transplant. Ann Clin Transl Neurol. 9, 206–220 10.1002/acn3.51510 35106961 PMC8862434

[110] Shin J.S., Subhan M.O., Cambridge G., Guo Y., de Groot R., Scully M. et al. (2022) Alterations in B- and circulating T-follicular helper cell subsets in immune thrombotic thrombocytopenic purpura. Blood Adv. 6, 3792–3802 10.1182/bloodadvances.2022007025 35507753 PMC9631570

[111] Fonseca V.R., Romão V.C., Agua-Doce A., Santos M., López-Presa D., Ferreira A.C. et al. (2018) The Ratio of Blood T Follicular Regulatory Cells to T Follicular Helper Cells Marks Ectopic Lymphoid Structure Formation While Activated Follicular Helper T Cells Indicate Disease Activity in Primary Sjögren's Syndrome. Arthritis Rheumatol. 70, 774–784 10.1002/art.40424 29361207

[112] Kim J.W., Lee J., Hong S.M., Lee J., Cho M.L. and Park S.H. (2019) Circulating CCR7loPD-1hi Follicular Helper T Cells Indicate Disease Activity and Glandular Inflammation in Patients with Primary Sjögren's Syndrome. Immune Netw. 19, e26 10.4110/in.2019.19.e26 31501714 PMC6722269

[113] Long Y., Zhao X., Liu C., Xia C. and Liu C. (2020) Activated inducible co-stimulator-positive programmed cell death 1-positive follicular helper T cells indicate disease activity and severity in ulcerative colitis patients. Clin. Exp. Immunol. 202, 106–118 10.1111/cei.13485 32621310 PMC7488121

[114] Yan X., Gu Y., Wang C., Sun S., Wang X., Tian J. et al. (2019) Unbalanced expression of membrane-bound and soluble inducible costimulator and programmed cell death 1 in patients with myasthenia gravis. Clin. Immunol. 207, 68–78 10.1016/j.clim.2019.07.011 31374257

[115] McCarty B., Mwamzuka M., Marshed F., Generoso M., Alvarez P., Ilmet T. et al. (2018) Low Peripheral T Follicular Helper Cells in Perinatally HIV-Infected Children Correlate With Advancing HIV Disease. Front Immunol. 9, 1901 10.3389/fimmu.2018.01901 30197641 PMC6117426

[116] Ricard L., Eshagh D., Siblany L., de Vassoigne F., Malard F., Laurent C. et al. (2022) 6-Sulfo LacNAc monocytes are quantitatively and functionally disturbed in systemic sclerosis patients. Clin. Exp. Immunol. 209, 175–181 10.1093/cei/uxac059 35758259 PMC9390843

[117] Lalinde-Ruiz N., Rodríguez I.J., Bernal-Estévez D.A. and Parra-López C.A. (2021) Young but not older adults exhibit an expansion of CD45RA+CCR7+CD95+ T follicular helper cells in response to tetanus vaccine. Exp. Gerontol. 156, 111599 10.1016/j.exger.2021.111599 34688830

[118] Lo Tartaro D., Neroni A., Paolini A., Borella R., Mattioli M., Fidanza L. et al. (2022) Molecular and cellular immune features of aged patients with severe COVID-19 pneumonia. Commun Biol. 5, 590 10.1038/s42003-022-03537-z 35710943 PMC9203559

[119] Teng F., Felix K.M., Bradley C.P., Naskar D., Ma H., Raslan W.A. et al. (2017) The impact of age and gut microbiota on Th17 and Tfh cells in K/BxN autoimmune arthritis. Arthritis Res. Ther. 19, 188 10.1186/s13075-017-1398-6 28810929 PMC5558662

[120] Gowthaman U., Chen J.S., Zhang B., Flynn W.F., Lu Y., Song W. et al. (2019) Identification of a T follicular helper cell subset that drives anaphylactic IgE. Science 365, eaaw6433 10.1126/science.aaw6433 31371561 PMC6901029

[121] Gryzik S., Hoang Y., Lischke T., Mohr E., Venzke M., Kadner I. et al. (2022) Identification of a super-functional Tfh-like subpopulation in murine lupus by pattern perception. Elife 9, e53226 10.7554/eLife.53226 PMC7274784 32441253

[122] Huang Q.Q., Hang Y., Doyle R., Mao Q., Fang D. and Pope R.M. (2023) Mechanisms regulating the loss of Tregs in HUPO mice that develop spontaneous inflammatory arthritis. iScience 26, 106734 10.1016/j.isci.2023.106734 37216119 PMC10193230

[123] Floudas A., Neto N., Orr C., Canavan M., Gallagher P., Hurson C. et al. (2023) Loss of balance between protective and pro-inflammatory synovial tissue T-cell polyfunctionality predates clinical onset of rheumatoid arthritis. Ann. Rheum. Dis. 81, 193–205 10.1136/annrheumdis-2021-220458 34598926

[124] Peng J., Lu X., Xie K., Xu Y., He R., Guo L. et al. (2019) Dynamic Alterations in the Gut Microbiota of Collagen-Induced Arthritis Rats Following the Prolonged Administration of Total Glucosides of Paeony. Front Cell Infect Microbiol. 9, 204 10.3389/fcimb.2019.00204 31245305 PMC6581682

